# Glucose-induced microRNA-218 suppresses the proliferation and promotes the apoptosis of human retinal pigment epithelium cells by targeting *RUNX2*

**DOI:** 10.1042/BSR20192580

**Published:** 2019-12-23

**Authors:** Rui Yao, Xiaoxi Yao, Ru Liu, Jingli Peng, Tao Tian

**Affiliations:** 1 Xiangya School of Medicine, Central South Unversity, Changsha, Hunan, China; 2 Department of Neurology, Chenzhou NO.1 People’s Hospital, Chenzhou, Hunan, China; 3 Department of Ophthalmology, Chenzhou NO.1 People’s Hospital, Chenzhou, Hunan, China

**Keywords:** diabetic retinopathy, glucose, human retinal pigment epithelium cells, microRNA-218, runt-related transcription factor 2

## Abstract

**Objective:** MicroRNA-218 (miR-218) critical for preventing the progression of numerous diseases, including diseases of the retinal pigment epithelium (RPE). However, the mechanism by which miR-218 regulates the PRE in humans remains largely unknown. Our study investigated the effects of glucose-induced miR-218 expression on human RPE cells (ARPE-19), as well as its targeted regulatory effect.

**Methods:** The levels of miR-218 and runt-related transcription factor 2 (RUNX2) expression were investigated by RT-qPCR or Western blot assays. Cell viability and apoptosis were assessed by CCK-8 assays, flow cytometry, and Hoechst staining. A luciferase reporter assay was performed to determine whether Runx2 is a target gene of miR-218.

**Results:** Our results showed that glucose up-regulated miR-218 expression, suppressed proliferation, and induced the apoptosis of ARPE-19 cells. We verified that miR-218 could inhibit the proliferation and facilitate the apoptosis of ARPE-19 cells, while inhibition of miR-218 expression produced the opposite effects. In terms of mechanism, we demonstrated that RUNX2 was a direct target of miR-218. Functional experiments showed that Runx2 served as a miR-218 target to help inhibit the proliferation and induction of apoptosis in ARPE-19 cells.

**Conclusion:** Our findings suggest the miR-218/Runx2 axis as a potential target for treating diabetic retinopathy (DR).

## Introduction

Diabetic retinopathy (DR) is a series of fundus lesions caused by abnormal retinal circulation resulting from aberrant glucose metabolism in diabetic patients [1]. In the early stage of DR, the basal membrane of capillary endothelial cells is thickened, peripheral cells are lost, and the automatic regulatory function of capillaries is decompensated. Later, endothelial cell function becomes impaired, blood components are exudated, and capillaries become occluded [2]. Extensive retinal ischemia causes retinal edema and neovascularization, complicated with vitreous hemorrhage, traction retinal detachment and many other complications that lead to loss of vision and even blindness [3,4]. DR is one of the most common causes of preventable blindness among working-age people, and the incidence of retinopathy in diabetic patients with a course > 10 years exceeds 50% [5]. DR can be divided into early stage and late stage DR based on its severity; proliferative diabetic retinopathy (PDR) represents the advanced stage, which can severely affect the vision of patients with diabetes [6,7]. DR is also recognized as a blinding eye disease. Therefore, it is of great importance to study its pathogenesis with the goal of preventing and treating DR, and finding new therapeutic molecular targets.

MicroRNAs (miRNAs) comprise a class of endogenous, tissue specific, and highly conserved RNAs with a length of 20–24 nucleotides [8]. MiRNA is a new type of gene expression regulatory factor that mainly binds to the 3’-untranslated region (UTR) of the target gene mRNA via complementary base pairs at the post-transcriptional level. This binding inhibits expression of the target mRNA, due to its degradation or lack of translation [9,10]. Studies have found that ∼90% of miRNAs in human eyes are highly selective and expressed in the retina and choroid [11,12]. Several studies have also verified that certain miRNAs including miR-126, miR-31, miR-200b, and miR-29 are closely associated with the progression of DR [13–17]. Research studies have found that miR-218 contributes to the clinical course of various diseases, including cancers [18–22], Hirschsprung’s disease [23], cardiomyocyte hypertrophy [24], neuropathic pain [25], and chronic obstructive pulmonary disease [26]. More importantly, recent studies have suggested that miR-218 is highly expressed in the crystalline lens of mice raised on a high glucose diet [27], and is associated with retinal neovascularization [28,29]. Therefore, we speculated that miR-218 might play a role in retinal diseases. However, the mechanism by which miR-218 functions in DR has not been fully elucidated.

In the present study, we further investigated the potential protective effect of miR-218 on human retinal pigment epithelial cells exposed to high glucose concentrations. We first examined how glucose and miR-218 effect the proliferation and apoptosis of ARPE-19 cells. In a mechanistic analysis, we used bioinformatics methods to predict potential biological targets for miR-218, and verified that runt-related transcription factor 2 (RUNX2) can function as a miR-218 sponge that is negatively regulated by miR-218. We also showed that RUNX2 could reverse the effects of miR-218 in ARPE-19 cells. Our findings suggest that miR-218 and RUNX2 might be vital targets for use in diagnosing and treating DR.

## Materials and methods

### Cell culture and treatment

ARPE-19 cells were purchased from ATCC (Manassas, VA, U.S.A.; CRL-2302) and maintained in DMEM/F12 medium (Life Technologies, Carlsbad, CA, U.S.A.; cat. no. 10565018) containing 10% fetal bovine serum (FBS, Gibco, Waltham, MA, U.S.A.; cat. no. 10091-148) and 1% penicillin–streptomycin (Gibco; cat. no. 15140-122). The cells were grown in a 37°C incubator with a 5% CO_2_ atmosphere. The ARPE-19 cells were treated with 5, 15, or 25 mM glucose or an equivalent amount of PBS for 0, 1, 2, and 3 days, respectively.

### Cell transfection

Negative control (NC) mimics, miR-218 mimics, a NC inhibitor, and a miR-218 inhibitor were purchased from GenePharma (Shanghai, China). A Runx2 overexpression plasmid and empty plasmid were purchased from Genechem (Shanghai, China). ARPE-19 cells (1 × 10^5^ cells/ml) were seeded into the wells of six-well plates and incubated for 8 h at 37°C. After incubation, the cells were transfected with NC mimics, miR-218 mimics, the NC inhibitor or miR-218 inhibitor for 24 h by using Lipofectamine 2000 (Invitrogen, Carlsbad, CA, U.S.A.) according to instructions provided by the manufacturer. Similarly, a Runx2 overexpression plasmid or empty plasmid was transfected into ARPE-19 cells by using Lipofectamine 2000 (Invitrogen) according to the manufacturer’s instructions.

### RNA extraction and quantitative real-time PCR (RT-qPCR) assay

Total RNA was extracted from the transfected ARPE-19 cells using TRIzol reagent (Takara, Japan; cat. no. 9109). cDNA was synthesized using an All-in-One™ First-Strand cDNA Synthesis Kit (GeneCopoeia, Rockville, MD, U.S.A.). The levels of RUNX2 and miR-218 expression were assessed by using SYBR-Green PCR Master Mix (Takara) on an ABI 7300 real time system. The results were analyzed using the 2^−△△*C*^_t_ method [30], and the sequences of primers are listed in Table 1.

**Table 1:** The PCR primers used in our study

ID	Sequence(5’- 3’)
GAPDH F	TGTTCGTCATGGGTGTGAAC
GAPDH R	ATGGCATGGACTGTGGTCAT
RUNX2 F	TGGTTACTGTCATGGCGGGTA
RUNX2 R	TCTCAGATCGTTGAACCTTGCTA
U6 F	CTCGCTTCGGCAGCACA
U6 R	AACGCTTCACGAATTTGCGT
All 1R	CTCAACTGGTGTCGTGGA
hsa-miR-218	UUGUGCUUGAUCUAACCAUGU
hsa-miR-218 RT	CTCAACTGGTGTCGTGGAGTCGGCAATTCAGTTGAGACATGGTT
hsa-miR-218 F	ACACTCCAGCTGGGTTGTGCTTGATCTAAC

### Western blot assay

Total proteins were obtained using an EpiQuik Total Histone Extraction Kit (Epigentek, Farmingdale, NY, U.S.A.; cat. no. OP-0006-100) according to instructions provided with the kit. Protein concentrations were determined using a Bradford Protein Assay Kit (Solarbio, Beijing, China; cat. no. PC0010). Aliquots of total protein from each sample were separated by 10% SDS-PAGE, and the protein bands were transferred onto PVDF membranes (Millipore, Burlington, MA, U.S.A.). The membranes were then blocked with 5% low fat dried milk for 2 h at room temperature and subsequently incubated with primary antibodies against RUNX2 (1:500, mouse, Abcam, Cambridge, U.K.; ab76956) and β-actin (1:3000, mouse, Abcam; ab20272) overnight at 4°C. After washing, the membranes were treated with Goat Anti-Mouse IgG (HRP, 1:5000, Abcam; ab205719) for 2 h at room temperature. The results were examined using enhanced chemiluminescent reagents.

### Cell Counting Kit-8 (CCK-8) assay

The proliferation of treated ARPE-19 cells was determined with the CCK-8 assay. Briefly, the treated ARPE-19 cells were seeded into 96-well plates at a concentration of 3000 cells/well, and maintained at 37°C for 8 h Next, a CCK-8 detection kit (Dojindo Molecular Technologies, Inc., Kumamoto, Japan) was used to measure cell proliferation at 0, 1, 2, and 3 days, respectively, according to instructions provided by the manufacturer.

### Hoechst staining

Apoptosis of the treated ARPE-19 cells was examined using Hoechst 33342 nuclear staining. For Hoechst staining, treated ARPE-19 cells were cultured for 30 min in six-well plates with 2 ml of medium that contained Hoechst 33342 blue fluorescent nuclear dye (Sigma-Aldrich, St. Louis, MO, U.S.A.). Nuclear morphology was detected by fluorescence microscopy that was performed using a Hoechst 33342 filter at 365 nm.

### Flow cytometry analysis

Cell apoptosis was evaluated using an Annexin V FITC/PI apoptosis detection kit (Abcam; ab14085). Treated ARPE-19 cells (2 × 10^6^ cells/ml) were harvested and incubated with 5 μl of Annexin V-FITC and 5 μl of PI for 15 min at room temperature in the dark. Cell apoptosis was examined using a BD FACSCalibur flow cytometer (Beckton Dickinson, Franklin Lakes, NJ, U.S.A.).

### Plasmid construction and dual luciferase activity assay

The 3’-UTRs of Runx2 cDNA containing the putative target and mutant sites for miR-218 were chemically generated and cloned into a pGL3 vector (Promega, Madison, WI, U.S.A.) to produce pGL3-Runx2-WT and pGL3-Runx2-Mut plasmids, respectively. ARPE-19 cells were plated into 24-well plates at a concentration of 2 × 10^5^ cells/well and cultured at 37°C for 24 h. Next, 200 ng of pGL3-Runx2-WT or pGL3-Runx2-Mut plasmids were co-transfected into ARPE-19 cells along with miR-218 mimics by using Lipofectamine 2000 (Invitrogen). Luciferase activity was then assessed using the Dual Luciferase Reporter Assay System (Promega). Firefly luciferase activity was normalized to that of Renilla luciferase activity.

### Statistical analysis

All data were analyzed by One-Way ANOVA performed with IBM SPSS 21.0 software (IBM Corp., Armonk, NY, U.S.A.). Experimental results are presented as the mean ± SD. A *P*-value < 0.05 was considered to be statistically significant.

## Results

### Glucose suppressed the proliferation and induced the apoptosis of ARPE-19 cells

**Figure 1:**
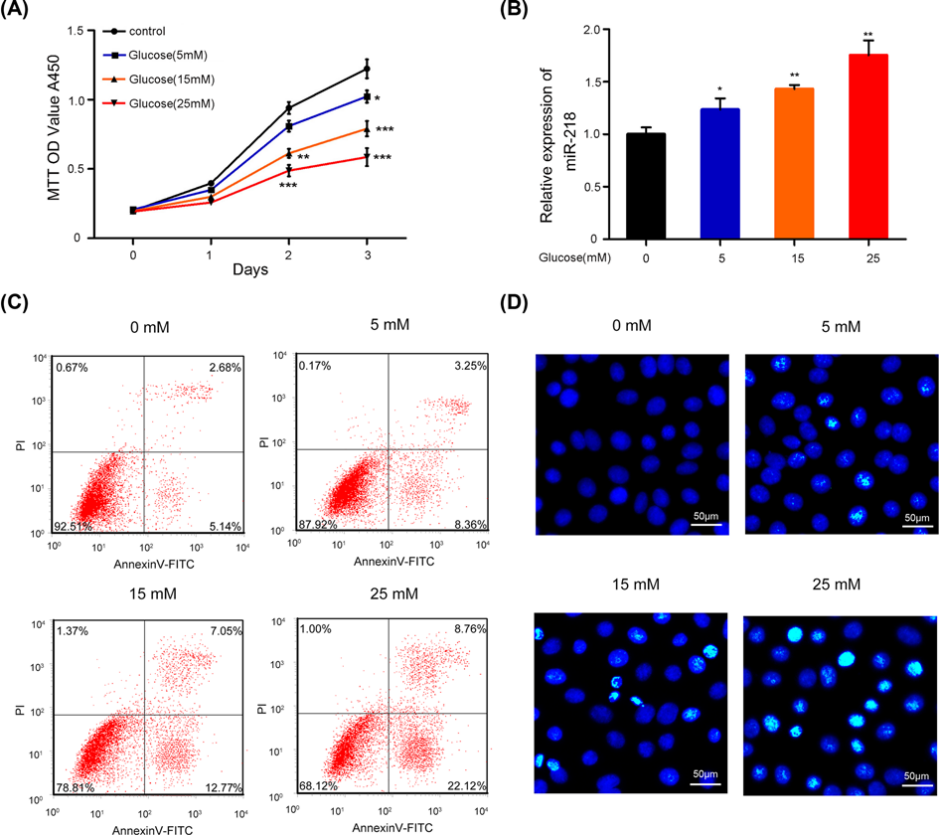
Glucose suppressed the proliferation and induced the apoptosis of ARPE-19 cells (**A**) The viability of ARPE-19 cells treated with PBS (control), 5, 15, or 25 mM glucose for 0, 1, 2, and 3 days, respectively, was checked using the CCK-8 assay. *P < 0.05, **P < 0.01, ***P < 0.001 versus control group. (**B**) The relative levels of miR-218 expression in ARPE-19 cells treated with glucose were analyzed by RT-qPCR. *P < 0.05, **P < 0.01 versus control group. (**C**) The apoptosis of ARPE-19 cells treated with different concentrations of glucose was analyzed by flow cytometry. (**D**) The effect of glucose on ARPE-19 cell apoptosis was investigated by Hoechst staining (original magnification ×200, scale bar = 50 μm). All experiments were repeated three times.

To explore whether glucose affected the proliferation and apoptosis of RPEs, ARPE-19 cells were treated with 0, 5, 15, or 25 mM glucose. CCK-8 analyses showed that cell proliferation was remarkably decreased among the glucose-treated ARPE-19 cells relative to the control cells, and the decrease was dose-dependent (*P* < 0.05, *P* < 0.01, *P* < 0.001, Figure 1A). In addition, we found that glucose significantly up-regulated miR-218 expression in ARPE-19 cells in a dose-dependent manner (*P* < 0.05, *P* < 0.01, Figure 1B). Moreover, Hoechst staining and flow cytometry analyses revealed that ARPE 19 cells treated with glucose had significantly increased rates of apoptosis when compared with control cells (Figure 1C,D). These results suggested that glucose inhibited the proliferation and promoted the apoptosis of ARPE-19 cells in dose dependent manners, which might be related to changes in miR-218 expression.

### Effects of miR-218 on the proliferation and apoptosis of ARPE-19 cells

**Figure 2:**
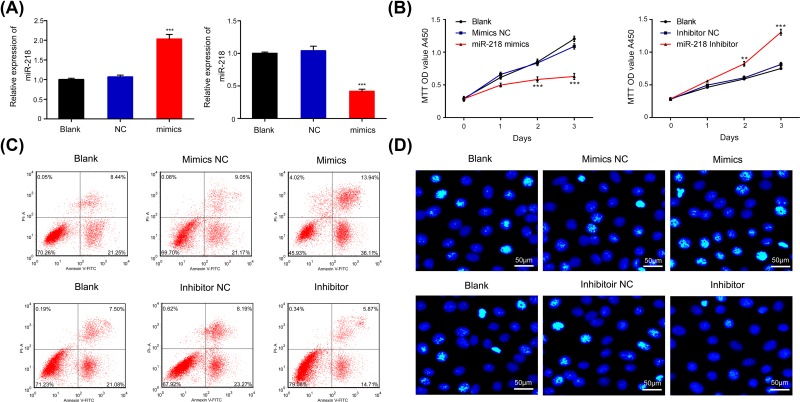
Effects of miR-218 on the proliferation and apoptosis of ARPE-19 cells ARPE-19 cells were transfected with NC mimics, miR-218 mimics, a NC inhibitor or miR-218 inhibitor, respectively. (**A**) The effects of transfection of ARPE-19 cells with the miR-218 mimics and inhibitor were confirmed by RT-qPCR; ***P < 0.001 vs. NC group. (**B**) CCK-8 analysis of cell proliferation among ARPE-19 cells transfected with miR-218 mimics or the inhibitor; **P < 0.01, ***P < 0.001 vs. NC group. (**C**) The apoptosis of transfected ARPE-19 cells was analyzed by flow cytometry. (**D**) Hoechst staining was used to evaluate the effects of miR-218 on the apoptosis of ARPE-19 cells transfected with miR-218 mimics or the inhibitor (original magnification ×200, scale bar = 50 μm); NC, negative control. All experiments were repeated three times.

To explore the impact of miR-218 on the proliferation and apoptosis of RPEs, ARPE-19 cells were transfected with miR-218 mimics to increase miR-218 expression or a miR-218 inhibitor to decrease miR-218 expression. Our results showed that miR-218 was significantly up-regulated in the miR-218 mimics group and significantly down-regulated in miR-218 inhibitor group when compared with miR-218 expression in a NC group (*P* < 0.001, Figure 2A). Subsequent CCK-8 assays verified that overexpression of miR-218 tended to reduce cell proliferation, while inhibition of miR-218 expression enhanced the proliferation of ARPE-19 cells (*P* < 0.01, *P* < 0.001, Figure 2B). Flow cytometry and Hoechst staining results showed that miR-218 overexpression promoted apoptosis, and miR-218 knockdown inhibited the apoptosis of ARPE-19 cells (Figure 2C,D). These results suggest that miR-218 can suppress the proliferation and facilitate the apoptosis of RPEs.

### MiR-218 negatively regulated Runx2 by targeted binding

**Figure 3:**
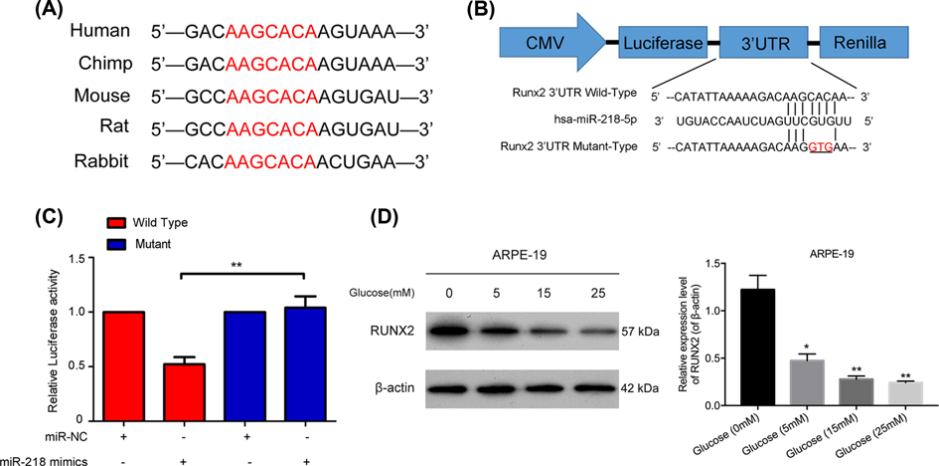
MiR-218 negatively regulated *Runx2* by targeted binding (**A**) The conserved sequence of the *Runx2* gene in humans, chimps, mice, rats, and rabbits. (**B**) The potential binding site between miR-218 and Runx2 mRNA was predicted by bioinformatics. (**C**) The relative levels of luciferase activity in ARPE-19 cells that were co-transfected with the Runx2 mutant or wild type plasmid and miR-218 mimics or its NC. **P < 0.01. (**D**) Runx2 protein expression in ARPE-19 cells treated with 0, 5, 15, or 25 mM glucose was examined by Western blotting. NC, negative control. All experiments were repeated three times.

Bioinformatics analysis results from TargetScan Human 5.1 (http://www.targetscan.org) predicted that *Runx2* might be the target gene for miR-218. We found that the *Runx2* gene was conserved in humans, chimps, mice, rats, and rabbits (Figure 3A). In order to determine whether Runx2 was a target gene of miR-218, we constructed a luciferase reporter vector containing the putative wild type or mutant Runx2 3’-UTR target site for miR-218 (Figure 3B). The WT-Runx2 or mutant-Runx2 vector was co-transfected into ARPE-19 cells along with miR-218 mimics. The relative levels of luciferase activity in cells co-transfected mutant Runx2 along with miR-218 mimics showed no obvious changes; however, a dramatic down-regulation of relative luciferase activity was observed in cells co-transfected with WT Runx2 and miR-218 mimics (*P* < 0.01, Figure 3C). These results indicated that glucose had downregulated Runx2 expression in the ARPE-19 cells in a dose-dependent manner (Figure 3D).

### Runx2 overexpression accelerated the proliferation and the suppressed apoptosis of ARPE-19 cells

**Figure 4:**
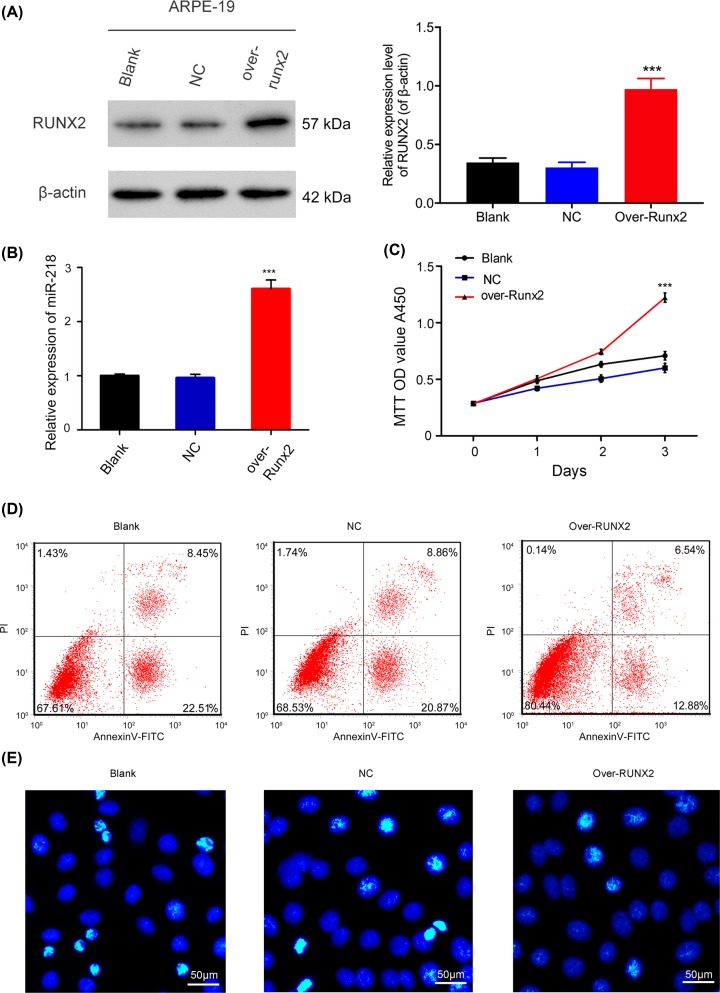
Effects of Runx2 overexpression on the proliferation and apoptosis of ARPE-19 cells ARPE-19 cells were transfected with the NC or Runx2 overexpression plasmid, respectively. The effect of transfection with the Runx2 overexpression plasmid was determined by Western blot (**A**) and RT-qPCR assays (**B**)**;** ***P < 0.001 vs. NC group. (**C**) CCK-8 analysis of the proliferation of ARPE-19 cells transfected with the Runx2 overexpression plasmid at 0, 1, 2, and 3 days, ***P < 0.001 vs. NC group. (**D**) The effect of Runx2 overexpression on the apoptosis of ARPE-19 cells as examined by flow cytometry. (**E**) The effect of Runx2 overexpression on the apoptosis of ARPE-19 cells as investigated by Hoechst staining (original magnification ×200, scale bar = 50 μm). All experiments were repeated three times.

Because *Runx2* was demonstrated to be a target gene for miR-218, we further evaluated whether Runx2 was involved in the proliferation and apoptosis of ARPE-19 cells. Results from RT-qPCR and Western blot assays revealed that Runx2 expression was higher in the Runx2 overexpression group than the NC group, indicating that the Runx2 overexpression plasmid had been successfully transfected into the ARPE-19 cells (*P* < 0.001, Figure 4A,B). CCK-8 assay results revealed that cells in the Runx2 overexpression group had higher proliferation rates than cells in the NC group (*P* < 0.001, Figure 4C). Hoechst staining and flow cytometry analyses showed that overexpression of Runx2 could significantly decrease the apoptosis of ARPE-19 cells (Figure 4D,E). In short, our results showed that Runx2 participated in the proliferation and apoptosis of ARPE-19 cells.

### MiR-218 suppressed the proliferation and induced the apoptosis of RPEs via Runx2

**Figure 5:**
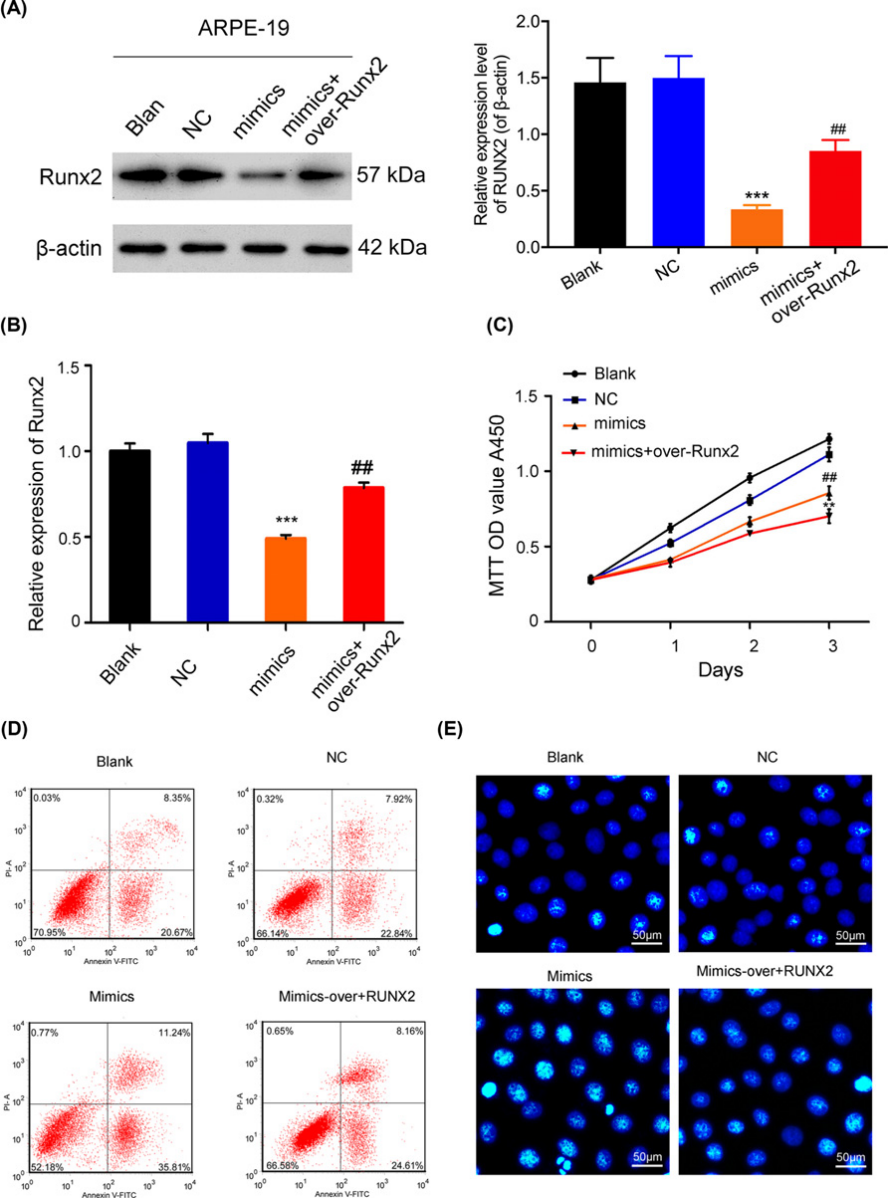
MiR-218 suppressed the proliferation and induced the apoptosis of RPEs via Runx2 ARPE-19 cells were either transfected with miR-218 mimics or co-transfected with miR-218 mimics or the Runx2 plasmid. (**A**) Western blot analysis of Runx2 expression; β-actin served as an internal control. (**B**) RT-qPCR analysis of Runx2 levels, ***P < 0.001 vs. NC group; ##*P* < 0.01 vs. mimics group. (**C**) Cell proliferation was examined by the CCK-8 assay, ***P < 0.001 vs. NC group; ##*P* < 0.01 vs. mimics group. (**D**) Cell apoptosis was assessed by flow cytometry. (**E**) Hoechst staining was performed to evaluate cell apoptosis (original magnification ×200, scale bar = 50 μm). All experiments were repeated three times.

To further verify whether miR-218 worked in conjunction with Runx2 to inhibit RPE cell proliferation and induce RPE cell apoptosis, ARPE-19 cells were co-transfected with miR-218 mimics and the Runx2 plasmid. Results showed that Runx2 overexpression markedly rescued the inhibition of Runx2 expression mediated by miR-218 (*P* < 0.01, *P* < 0.001, Figure 5A,B). CCK-8 assays indicated that cell proliferation was significantly reduced in the miR-218 mimics group, while that effect was reversed when miR-218 mimics and the Runx2 overexpression plasmid were co-transfected (*P* < 0.01, *P* < 0.001, Figure 5C). Regarding apoptosis, Runx2 overexpression dramatically reversed the promotion of cell apoptosis induced by miR-218 (Figure 5D,E). When taken together, these results proved that overexpression of Runx2 could abolish the effects of miR-218 on the proliferation and apoptosis of RPE cells.

## Discussion

DR is one of the principal diabetic microvascular diseases, and a type of fundus disease that has become the leading cause of blindness in diabetic patients [31]. Human retinal pigment epithelial cells (RPEs), such as ARPE-19 cells, are located between the vascular-rich choroid and the retinal nerve cortex, which are crucial nutrient, maintenance, and metabolic tissues of the retina [32]. RPEs are easily stimulated due to their special position and function, and this stimulation can lead to multiple retinopathies [33]. In our study, glucose was utilized to induce ARPE-19 cells, and we verified that high glucose concentrations could inhibit the proliferation and induce the apoptosis of ARPE-19 cells.

It is well known that miRNAs significantly affect many biological processes, such as cell proliferation, apoptosis, differentiation, and metabolism [34,35]. An increasing body of evidence indicates that miR-218 is downregulated in various diseases. For example, down-regulation of miR-218 was reported to result in epithelial–mesenchymal transition and the metastasis of lung cancer [21]. Other studies indicated that miR-218 could prevent the cell cycle progression of gastric cancer cells [18], participate in cardiomyocyte hypertrophy via REST [24], and affect chronic obstructive pulmonary disease (COPD) by regulating the TNFR1-mediated activation of NF-κB [26]. Research has also verified that miRNAs can significantly contribute to endothelial dysfunction [36,37]. Regarding miR-218, studies have suggested that miR-218 can suppress oxygen-induced retinal neovascularization [29], and is involved in retinal neovascularization [28]. In our study, we proved that glucose could increase miR-218 expression in ARPE-19 cells. Therefore, we speculated that miR-218 might be involved in the glucose regulation of SW480 cell function.

Furthermore, we also verified that up-regulation of miR-218 could inhibit the proliferation and increase the apoptosis of ARPE-19 cells, while down-regulation of miR-218 could promote the proliferation and suppress the apoptosis of ARPE-19 cells. Therefore, our data further suggest miR-218 as a therapeutic target for DR.

The transcription factor Runx2 is a member of the RUNX transcription factor family [38]. The RUNX family comprises transcription factor proteins that are responsible for encoding a class of Runt DNA-binding domain nucleoproteins [39,40]. This DNA binding domain is composed of 128 amino acids and is highly homologous with the drosophila gene *Runt* in the evolutionary process; therefore, it is called the Runt domain [41]. The RUNX family of transcription factors can bind to the core binding factor β (CBFβ) to form a heterodimer that greatly enhances their ability to bind to DNA [42]. Numerous studies have demonstrated that RUNX2 participates in the physiological and pathological processes of various diseases, such as cancers [43–45], asthma [46], cleidocranial dysplasia [47,48], and acromegaly [49]. Many studies have also verified that RUNX2 expression is closely associated with the progression of various diseases by affecting cell proliferation, apoptosis, autophagy, metastasis, and osteogenic differentiation [50–52]. However, its function in RPE cells has remained unknown. In the present study, we proved for the first time that overexpression of Runx2 could dramatically accelerate the proliferation and suppress the apoptosis of ARPE-19 cells. A previous study showed that human RPE cells could be activated to become self-renewing cells *in vitro*, and Runx2, acted as an early definitive marker of osteogenic differentiation marker, and was involved with RPE plasticity [53]. Therefore, we demonstrated that Runx2 could be involved in the biological processes of human RPE cells.

Furthermore, to explore the possible regulatory mechanism of miR-218 in RPEs, the target genes of miR-218 were predicted using bioinformatics analysis we also verified that *Runx2* is a target gene of miR-218, and is negatively regulated by miR-218. Moreover, we revealed that Runx2 overexpression could dramatically reverse the effect that miR-218 exerted on ARPE-19 cell proliferation and apoptosis, suggesting that miR-218 suppresses the proliferation and induces the apoptosis of RPEs by targeting *Runx2*. However, our study has many limitations. For example, *in vivo* experiments should be performed to further verify our findings, and the roles played by the miR-218/Runx2 axis need to be investigated in hyperglycemic cell models.

## Conclusion

We proved that miR-218 levels can be up-regulated by glucose in ARPE-19 cells. In addition, *Runx2* was identified as a target gene of miR-218, and miR-218 was shown to inhibit the proliferation and accelerate the apoptosis of ARPE-19 cells by targeting *Runx2*. Therefore, we for the first time verified that miR-218 plays essential roles in inhibiting the proliferation and promoting the apoptosis of RPEs by targeting *RUNX2*, suggesting the miR-218/RUNX2 axis as a therapeutic target for RD.

## Abbreviations

CBFβ: core binding factor β
COPD: chronic obstructive pulmonary disease
DR: diabetic retinopathy
miR-218: microRNA-218
PDR: proliferative diabetic retinopathy
RPE: retinal pigment epithelium
RUNX2: runt-related transcription factor 2

## References

[1] Morrison J.L. et al. (2016) Diabetic retinopathy in pregnancy: a review. Clin. Exp. Ophthalmol. 44, 321–334 10.1111/ceo.12760 27062093

[2] Wong T.Y. et al. (2016) Diabetic retinopathy. Nat. Rev. Dis. Primers. 2, 16012 10.1038/nrdp.2016.12 27159554

[3] Roy S. et al. (2017) Mechanistic Insights into Pathological Changes in the Diabetic Retina: Implications for Targeting Diabetic Retinopathy. Am. J. Pathol. 187, 9–19 10.1016/j.ajpath.2016.08.022 27846381 PMC5225303

[4] Traveset A. et al. (2016) Lower Hemoglobin Concentration Is Associated with Retinal Ischemia and the Severity of Diabetic Retinopathy in Type 2 Diabetes. J. Diabetes Res. 2016, 3674946 10.1155/2016/3674946 27200379 PMC4855016

[5] Liew G., Michaelides M. and Bunce C. (2014) A comparison of the causes of blindness certifications in England and Wales in working age adults (16-64 years), 1999-2000 with 2009-2010. BMJ Open 4, e004015 10.1136/bmjopen-2013-004015 24525390 PMC3927710

[6] Hollanders K. et al. (2017) AMA0428, A Potent Rock Inhibitor, Attenuates Early and Late Experimental Diabetic Retinopathy. Curr. Eye Res. 42, 260–272 10.1080/02713683.2016.1183030 27399806

[7] Jenkins A.J. et al. (2015) Biomarkers in Diabetic Retinopathy. Rev. Diabet. Stud. 12, 159–195 10.1900/RDS.2015.12.159 26676667 PMC5397989

[8] Cui J. et al. (2017) Nutrition, microRNAs, and Human Health. Adv. Nutr. 8, 105–112 10.3945/an.116.013839 28096131 PMC5227982

[9] Tufekci K.U. et al. (2014) The role of microRNAs in human diseases. Methods Mol. Biol. 1107, 33–50 10.1007/978-1-62703-748-8_3 24272430

[10] Miura K. et al. (2018) Expression levels of C19MC and C14MC microRNAs in complete hydatidiform moles and ovarian mature cystic teratomas. Eur. J. Gynaecological Oncol. 39, 277–280

[11] Karali M. et al. (2016) High-resolution analysis of the human retina miRNome reveals isomiR variations and novel microRNAs. Nucleic Acids Res. 44, 1525–1540 10.1093/nar/gkw039 26819412 PMC4770244

[12] Pandi S.P.S. et al. (2013) Extremely complex populations of small RNAs in the mouse retina and RPE/choroid. Biochem. Mol. Biol 54, 8140–8151 10.1167/iovs.13-12631 24235016

[13] Mitra R.N. et al. (2016) Nanoparticle-mediated miR200-b delivery for the treatment of diabetic retinopathy. J. Control. Release 236, 31–37 10.1016/j.jconrel.2016.06.020 27297781 PMC5328608

[14] Dantas da Costa E.S.M.E. et al. (2019) Plasma levels of miR-29b and miR-200b in type 2 diabetic retinopathy. J. Cell. Mol. Med. 23, 1280–1287 10.1111/jcmm.14030 30467971 PMC6349208

[15] Qin L.L. et al. (2017) MicroRNA-126: a promising novel biomarker in peripheral blood for diabetic retinopathy. Int. J. Ophthalmol. 10, 530–534 28503423 10.18240/ijo.2017.04.05 PMC5406628

[16] Rovira-Llopis S. et al. (2018) Downregulation of miR-31 in Diabetic Nephropathy and its Relationship with Inflammation. Cell. Physiol. Biochem. 50, 1005–1014 10.1159/000494485 30355913

[17] Zhao B.W. et al. (2019) MiR-29 regulates retinopathy in diabetic mice via the AMPK signaling pathway. Eur. Rev. Med. Pharmacol. Sci. 23, 3569–3574 31114980 10.26355/eurrev_201905_17778

[18] Deng M. et al. (2017) miR-218 suppresses gastric cancer cell cycle progression through the CDK6/Cyclin D1/E2F1 axis in a feedback loop. Cancer Lett. 403, 175–185 10.1016/j.canlet.2017.06.006 28634044

[19] Han M., Chen L. and Wang Y. (2018) miR-218 overexpression suppresses tumorigenesis of papillary thyroid cancer via inactivation of PTEN/PI3K/AKT pathway by targeting Runx2. Onco. Targets Ther. 11, 6305–6316 10.2147/OTT.S172152 30319270 PMC6167989

[20] Lun W. et al. (2018) MiR-218 regulates epithelial-mesenchymal transition and angiogenesis in colorectal cancer via targeting CTGF. Cancer Cell Int. 18, 83 10.1186/s12935-018-0575-2 29977158 PMC5994014

[21] Shi Z.M. et al. (2017) Downregulation of miR-218 contributes to epithelial-mesenchymal transition and tumor metastasis in lung cancer by targeting Slug/ZEB2 signaling. Oncogene 36, 2577–2588 10.1038/onc.2016.414 28192397 PMC5422710

[22] Wang T. et al. (2017) MiR-218 suppresses the metastasis and EMT of HCC cells via targeting SERBP1. Acta. Biochim. Biophys. Sin. (Shanghai) 49, 383–391 10.1093/abbs/gmx017 28369267

[23] Tang W. et al. (2015) SLIT2/ROBO1-miR-218-1-RET/PLAG1: a new disease pathway involved in Hirschsprung’s disease. J. Cell. Mol. Med. 19, 1197–1207 10.1111/jcmm.12454 25786906 PMC4459835

[24] Liu J.J. et al. (2016) miR-218 Involvement in Cardiomyocyte Hypertrophy Is Likely through Targeting REST. Int. J. Mol. Sci. 17, 848–858 10.3390/ijms 27258257 PMC4926382

[25] Li L. and Zhao G. (2016) Downregulation of microRNA-218 relieves neuropathic pain by regulating suppressor of cytokine signaling 3. Int. J. Mol. Med. 37, 851–858 10.3892/ijmm.2016.2455 26782075

[26] Xu H. et al. (2017) MicroRNA-218 acts by repressing TNFR1-mediated activation of NF-kappaB, which is involved in MUC5AC hyper-production and inflammation in smoking-induced bronchiolitis of COPD. Toxicol. Lett. 280, 171–180 10.1016/j.toxlet.2017.08.079 28864214

[27] Raghunath A. and Perumal E. (2015) Micro-RNAs and their roles in eye disorders. Ophthalmic. Res. 53, 169–186 10.1159/000371853 25832915

[28] Kong Y. et al. (2016) Slit-miR-218-Robo axis regulates retinal neovascularization. Int. J. Mol. Med. 37, 1139–1145 10.3892/ijmm.2016.2511 26935869

[29] Han S. et al. (2016) microRNA-218 Inhibits Oxygen-induced Retinal Neovascularization via Reducing the Expression of Roundabout 1. Chin. Med. J. (Engl.) 129, 709–715 10.4103/0366-6999.178013 26960375 PMC4804418

[30] Livak K.J. and Schmittgen T.D. (2001) Analysis of relative gene expression data using real-time quantitative PCR and the 2(-Delta Delta C(T)) Method. Methods 25, 402–408 10.1006/meth.2001.1262 11846609

[31] Antonetti D.A. et al. (2006) Diabetic retinopathy: seeing beyond glucose-induced microvascular disease. Diabetes 55, 2401–2411 10.2337/db05-1635 16936187

[32] Garcia-Ramírez M. et al. (2010) Measuring permeability in human retinal epithelial cells (ARPE-19): implications for the study of diabetic retinopathy. Methods Mol. Biol. 763, 179–194 10.1007/978-1-61779-191-8_12 21874452

[33] Stern J. and Temple S. (2015) Retinal pigment epithelial cell proliferation. Exp. Biol. Med. (Maywood) 240, 1079–1086 10.1177/1535370215587530 26041390 PMC4935281

[34] Ding T. et al. (2017) Predicting microRNA biological functions based on genes discriminant analysis. Comput. Biol. Chem. 71, 230–235 10.1016/j.compbiolchem.2017.09.008 29033260

[35] Liu B., Li J. and Cairns M.J. (2014) Identifying miRNAs, targets and functions. Brief. Bioinform. 15, 1–19 10.1093/bib/bbs075 23175680 PMC3896928

[36] Nemecz M. et al. (2016) Role of MicroRNA in Endothelial Dysfunction and Hypertension. Curr. Hypertens. Rep. 18, 87 10.1007/s11906-016-0696-8 27837398 PMC7102349

[37] Santulli G. (2016) MicroRNAs and Endothelial (Dys) Function. J. Cell. Physiol. 231, 1638–1644 10.1002/jcp.25276 26627535 PMC4871250

[38] Morita K. et al. (2017) Genetic regulation of the RUNX transcription factor family has antitumor effects. J. Clin. Invest. 127, 2815–2828 10.1172/JCI91788 28530640 PMC5490777

[39] Cunningham M.L. et al. (2010) Cleidocranial dysplasia with severe parietal bone dysplasia: C‐terminal RUNX2 mutations. Birth Defects Res. A Clin. Mol. Teratol. 76, 78–85 10.1002/bdra.20231 16463420

[40] Komori T. (2018) Runx2, an inducer of osteoblast and chondrocyte differentiation. Histochem. Cell Biol. 149, 313–323 10.1007/s00418-018-1640-6 29356961

[41] Deltcheva E. and Nimmo R. (2017) RUNX transcription factors at the interface of stem cells and cancer. Biochem. J. 474, 1755–1768 10.1042/BCJ20160632 28490659

[42] Illendula A. et al. (2016) Small Molecule Inhibitor of CBFbeta-RUNX Binding for RUNX Transcription Factor Driven Cancers. EBioMedicine 8, 117–131 10.1016/j.ebiom.2016.04.032 27428424 PMC4919611

[43] Gowda P.S. et al. (2018) Runx2 Suppression by miR-342 and miR-363 Inhibits Multiple Myeloma Progression. Mol. Cancer Res. 16, 1138–1148 10.1158/1541-7786.MCR-17-0606 29592898 PMC6030427

[44] Lu H. et al. (2018) RUNX2 Plays An Oncogenic Role in Esophageal Carcinoma by Activating the PI3K/AKT and ERK Signaling Pathways. Cell. Physiol. Biochem. 49, 217–225 10.1159/000492872 30138923

[45] Yamada D. et al. (2018) RUNX2 Promotes Malignant Progression in Glioma. Neurochem. Res. 43, 2047–2054 10.1007/s11064-018-2626-4 30203400

[46] Shi N., Zhang J. and Chen S.Y. (2017) Runx2, a novel regulator for goblet cell differentiation and asthma development. FASEB J. 31, 412–420 10.1096/fj.201600954R 27825108 PMC5161525

[47] Zeng L. et al. (2017) Functional analysis of novel RUNX2 mutations in cleidocranial dysplasia. Mutagenesis 32, 437–443 10.1093/mutage/gex012 28505335

[48] Zeng L. et al. (2018) A novel 18-bp in-frame deletion mutation in RUNX2 causes cleidocranial dysplasia. Arch. Oral. Biol. 96, 243–248 10.1016/j.archoralbio.2017.10.020 29089101

[49] Valenti M.T. et al. (2018) Runx2 overexpression compromises bone quality in acromegalic patients. Endocr. Relat. Cancer 25, 269–277 10.1530/ERC-17-0523 29295822

[50] Sancisi V. et al. (2017) RUNX2 expression in thyroid and breast cancer requires the cooperation of three non-redundant enhancers under the control of BRD4 and c-JUN. Nucleic Acids Res. 45, 11249–11267 10.1093/nar/gkx802 28981843 PMC5737559

[51] Tandon M. et al. (2018) The role of Runx2 in facilitating autophagy in metastatic breast cancer cells. J. Cell. Physiol. 233, 559–571 10.1002/jcp.25916 28345763

[52] Vimalraj S. et al. (2015) Runx2: Structure, function, and phosphorylation in osteoblast differentiation. Int. J. Biol. Macromol. 78, 202–208 10.1016/j.ijbiomac.2015.04.008 25881954

[53] Salero E. et al. (2012) Adult human RPE can be activated into a multipotent stem cell that produces mesenchymal derivatives. Cell Stem Cell 10, 88–95 10.1016/j.stem.2011.11.018 22226358

